# Diabetes in Sub Saharan Africa 1999-2011: Epidemiology and public health implications. a systematic review

**DOI:** 10.1186/1471-2458-11-564

**Published:** 2011-07-14

**Authors:** Victoria Hall, Reimar W Thomsen, Ole Henriksen, Nicolai Lohse

**Affiliations:** 1 Freelance Public Health Research Consultant, Private Practice, London, UK; 2 Department of Clinical Epidemiology, Clinical Institute, Aarhus University Hospital, DK-8200 Aarhus N, Denmark; 3 Senior Economist, Global Stakeholder Engagement, NovoNordisk A/S, Novo Allé, DK-2880 Bagsværd, Denmark; 4 Programme Director, Global Stakeholder Engagement, NovoNordisk A/S, Novo Allé, DK-2880 Bagsværd, Denmark

## Abstract

**Background:**

Diabetes prevalence is increasing globally, and Sub-Saharan Africa is no exception. With diverse health challenges, health authorities in Sub-Saharan Africa and international donors need robust data on the epidemiology and impact of diabetes in order to plan and prioritise their health programmes. This paper aims to provide a comprehensive and up-to-date review of the epidemiological trends and public health implications of diabetes in Sub-Saharan Africa.

**Methods:**

We conducted a systematic literature review of papers published on diabetes in Sub-Saharan Africa 1999-March 2011, providing data on diabetes prevalence, outcomes (chronic complications, infections, and mortality), access to diagnosis and care and economic impact.

**Results:**

Type 2 diabetes accounts for well over 90% of diabetes in Sub-Saharan Africa, and population prevalence proportions ranged from 1% in rural Uganda to 12% in urban Kenya. Reported type 1 diabetes prevalence was low and ranged from 4 per 100,000 in Mozambique to 12 per 100,000 in Zambia. Gestational diabetes prevalence varied from 0% in Tanzania to 9% in Ethiopia. Proportions of patients with diabetic complications ranged from 7-63% for retinopathy, 27-66% for neuropathy, and 10-83% for microalbuminuria. Diabetes is likely to increase the risk of several important infections in the region, including tuberculosis, pneumonia and sepsis. Meanwhile, antiviral treatment for HIV increases the risk of obesity and insulin resistance. Five-year mortality proportions of patients with diabetes varied from 4-57%. Screening studies identified high proportions (> 40%) with previously undiagnosed diabetes, and low levels of adequate glucose control among previously diagnosed diabetics. Barriers to accessing diagnosis and treatment included a lack of diagnostic tools and glucose monitoring equipment and high cost of diabetes treatment. The total annual cost of diabetes in the region was estimated at US$67.03 billion, or US$8836 per diabetic patient.

**Conclusion:**

Diabetes exerts a significant burden in the region, and this is expected to increase. Many diabetic patients face significant challenges accessing diagnosis and treatment, which contributes to the high mortality and prevalence of complications observed. The significant interactions between diabetes and important infectious diseases highlight the need and opportunity for health planners to develop integrated responses to communicable and non-communicable diseases.

## Background

Sub-Saharan Africa, like the rest of the world, is experiencing an increasing prevalence of diabetes alongside other non-communicable diseases [1]. In 2010 12.1 million people were estimated to be living with diabetes in Africa, and this is projected to increase to 23.9 million by 2030 [2]. In Sub-Saharan Africa this trend is emerging in a region grappling with high rates of communicable diseases - including the highest global prevalence of HIV [3], Tuberculosis [4] and Malaria [5]. Diabetes is a component cause of several other important and often lethal diseases, both non-communicable diseases such as cardiovascular disease [6] and renal disease [7], and communicable diseases such as pneumonia [8], bacteraemia [9,10] and tuberculosis [11], which have considerable impacts on morbidity and mortality in the region [12-17]. With this double burden of disease and limited resources, diabetes must compete for political attention and financial investment. With various regional and international meetings and initiatives [18], including the UN high-level meeting on Non-communicable Diseases scheduled for 19-20 September 2011, diabetes and other non-communicable diseases are beginning to get greater political attention. Policymakers need guidance from strong reviews of current information on trends and public health impact. This review expands on recent reviews of diabetes in the region [19-21], providing greater consideration of the diabetes prevalence, access to diabetes diagnosis and care, clinical outcomes following diabetes (chronic diabetes complications, infections and mortality), and economic costs.

## Methods

We conducted a systematic review of all papers published on diabetes in Sub-Saharan Africa between January 1999 and March 2011 and available on PubMed. We defined Sub-Saharan Africa as all mainland African countries south of the Sahara with the addition of the island state of Madagascar. We searched for articles providing data from this region on diabetes prevalence, diabetes outcomes (chronic diabetes complications, infections, and mortality), access to diabetes diagnosis and care, and the economic burden caused by diabetes. A combined keyword search on PubMed identified 1102 papers. See Additional File 1: Annex 1 for a description of the review and Additional File 2: Annex 2 for the keywords used. The references of included articles were scanned to identify additional articles of interest published before January 1999. Grey literature, from sources including the websites of the World Bank, World Health Organisation and International Diabetes Federation was also reviewed.

## Results

### The prevalence of diabetes in Sub-Saharan Africa

Type 2 diabetes accounts for over 90% of diabetes cases in Sub-Saharan Africa [19], whilst Type 1 diabetes, gestational diabetes, and variant forms such as atypical 'ketosis-prone' diabetes and malnutrition-related diabetes constitute the remainder.

#### Type 2 diabetes

Just nine countries in the region have reported data from type 2 diabetes (T2DM) prevalence surveys in the last decade (see table 1) [22-36]. Two of these countries have conducted population surveys with the assistance of the World Health Organisation's 'STEPwise Approach to Chronic Disease Surveillance Management'. Prevalence in the general population of T2DM recorded in these studies ranged from 0.6% in rural Uganda [35] to 12% in urban Kenya [27]. A low to medium prevalence (0-7%) was recorded in Cameroon, Ghana, Guinea, Kenya, Nigeria, South Africa and Uganda and a very high prevalence (> 10%) was recorded in Zimbabwe (table 1).

**Table 1:** Prevalence of Type II Diabetes in cross sectional surveys in Sub-Saharan Africa 1999-2011

Country	Author	Site	Sample size (Participation rate %)	Age range	Method	Prevalence (%) (95% CI)			Un-diagnosed diabetes (%)	Prevalence IGT (%) (95% CI)	Prevalence IFG (%) (95% CI)	% Obese (BMI ≥30)	
										
						M	F	M+F				Males	Females
**Cameroon**	Mbanya 1999[22]	Urban & Rural	Urban: 295 (> 90) Rural: 384 (> 90)	24-74	OGTT/FBG (WHO 85, ADA 97)	Urban: 1.0* (0.1-3.6) Rural: 1.1* (0.1-4.0)	Urban: 2.8* (0.9-6.6) Rural: 0.5* (0-3.0)	Urban: 2.0* Rural: 0.8*	-	Urban (M: 1.6* (0.3-4.6) F: 4.6* (0.8-13)) Rural: (M: 6.4 (3.3-11.3) F: 3.1* (1.1-6.8))		-	-
	Sobngwi 2002[23]	Urban & Rural	Urban: 1183 (96) Rural: 1282 (98)	≥15	FBG (WHO 98)	Urban: 6.2* (3.7-8.9) Rural: 4.7* (2.5-6.9)	Urban: 4.7* (2.6-6.8) Rural: 2.9* (1.5-4.4)	-	-	-	Urban: (M: 4.4 (2.6-4.3) F: 10.4 (7.8-13)) Rural: (M: 4.9 (2.4-7.4) F: 4.7 (2.8-6.6))	Urban: 5.4* Rural: 0.5*	Urban: 17.1* Rural: 3.0*
	MOH Cambod 2004 [24]	Urban	10,824	≥15	OGTT	6.40*	5.70*	6.06*	80	-	-	7.51*	21.25*

**Ghana**	Amoah 2002 [25]	Urban	4733 (75)	≥25	OGTT (WHO 98, ADA 97)	7.7*	5.5*	6.4*	69.9	10.7*	6.0*	-	-

**Guinea**	Balde 2007 [26]	Urban & Rural	1537 (77)	≥35	FBG (WHO 99)	-	-	Urban: 6.7* (5.1-8.3) Rural: 5.3* (3.6-7.0)	Urban: 59 Rural: 100	-	Urban: 10.3 (8.3-12.3) Rural: 17.7 (14.7-20.6)	-	-

**Kenya**	Christensen 2009 [27]	Urban & rural	Urban: 281 (98.2) Rural: 1178 (98.2)	≥17	FBG/OGTT (WHO 99)	Urban & Rural: 4.5* (2.0-10.2)	Urban & Rural: 4.2* (1.409.4)	Urban: 12.2* (5.4-23.2) Rural: 2.2* (0.8-5.2) Combined: 4.2* (2.0-7.7)	Urban: 21 Rural: 48	Urban: 13.2* (4.6-26.5) Rural: 8.6* (5.1-14.0) Combined: 12.0* (9.2-15.2)	-	-	-
	Mathenge 2010 [28]	Urban & Rural	Urban: 1437 Rural: 2959	≥50	RBG	-	-	**Urban: 10** **Rural: 5**	Urban: 23 Rural: 41	-	-	Urban: 20 Rural: 10	

**Nigeria**	Okesina 1999 [121]	Rural	500	> 40	FBG			**2.6**				-	-
	Nyenwe 2003 [29]	Urban	502 (67.1)	≥40	OGTT (WHO 99)	9.1*	6.3*	7.9*	41	-	-	-	-
	Oladapo 2010 [30]	Rural	2000	18-64	FBG	2.1	2.8	**2.5**	27	-	-	3.9	

**South Africa**	Erasmus 2001 [122]	Urban	374 (workers)	> 20	OGTT	-	-	4.5*				-	-
	Alberts 2005 [32]	Rural	2106	> 30	FBG	**-**	-	**8.8**				30	7.4
	Motala 2008 [33]	Rural	1025 (78.9)	> 15	OGTT (WHO 98)	3.5*	3.9*	3.9*	84.8	4.8*	41.5*	-	-

**Tanzania**	Aspray 2000 [34]	Urban & Rural	Urban: 770 Rural: 928	> 15	FBG (WHO 98)	Urban: 5.9* Rural: 1.7*	Urban: 5.7* Rural: 1.1*	-	-	-	Urban: (M: 3.6* W:4.7*) Rural: (M: 0.8* W: 1.1*)	Urban: 7.3* Rural: 0.2*	Urban: 19.8* Rural: 4.2*

**Uganda**	Maher 2010 [35]	Rural	6678	≥13	RPG	**-**	**-**	0.6* (0.4-0.7)	74	-	-	0.5	3.9

**Zimbabwe**	MOH STEPS 2005 [36]	Pop Rep	3081	≥25	FBG	**-**	**-**	**10**	-	M: 5.3 (3.3-8) F: 5.2 (4.1-6.7)	-	3.9	10.4

Method: OGTT - Oral Glucose Tolerance Test, FBG - Fasting Blood Glucose Test, WHO 85/97/99: World Health Organisation 1985/98/99 diagnostic criteria, ADA97/04 American Diabetes Association 1997/2004 diagnostic criteria; RPG - Random Plasma Glucose Test for "probable diabetes" (>11mmol/l), RBG - Random Blood Glucose Test diabetes categorised as >11.1mmol/l; *age standardised; IGT: Impaired Glucose Tolerance; IFG Impaired Fasting Glucose; Pop rep - sample representative of national population.

Variation in prevalence recorded within countries was common. Prevalence estimates varied considerably between different studies for some countries, with estimates for rural South Africa ranging from 3.9% [31] to 8.8% [33]. Variation between urban and rural populations was frequently observed, with a higher prevalence recorded in urban populations [22,23,26,27,33,34]. Prevalence recorded in Christensen's Kenyan survey ranged from 2% in rural areas to 12% in urban areas [27].

One recent study investigated the incidence of type 2 diabetes in Kinshasa among a cohort of 807 people, aged over 40 at baseline [37]. 93 participants developed T2DM during the study period (December 2004 to September 2008), corresponding to an incidence rate of 29 (95% CI 15-43) per 1,000 person-years.

#### Type 1 diabetes

Four studies estimating the prevalence and/or incidence of type 1 diabetes in the region were published since 1990 [38-41] (see table 2). Observed prevalence ranged from 3.5 per 100,000 persons in Mozambique [40], to 12 per 100,000 persons in Zambia [40]. Recorded incidence ranged from 1.5 per 100,000 persons per year in Tanzania [41] to 2.1 per 100,000 persons per year in Ethiopia [39].

**Table 2:** Prevalence and Incidence of Type 1 diabetes in Sub-Saharan Africa 1990-2011

Country	Author	Sample	Age	Incidence per 100,000 persons (95% CI)	Prevalence per 100,000 persons
Ethiopia	Aleumu (2009) [39]	1029	-	2.1 (2.0 - 2.2)	-

Mozambique	Beran (2005) [40]	-	0-19	-	3.5

Sudan	Elamin (1992) [38]	42,981	7-14	-	9.5

Tanzania	Swai (1993) [41]	86	0-19	1.5 (1.3 - 1.7)	-

Zambia	Beran (2005) [40]	-	0-19	-	12

#### Gestational diabetes mellitus

The literature review identified two studies on the prevalence of gestational diabetes in Sub-Saharan Africa 1999-present, one in Ethiopia [42] and one in South Africa [43] (see table 3). Three other relatively recent studies, published before January 1999, were identified [44-46]. The range of prevalence recorded in these five studies is considerable, from 0% among pregnant women in Tanzania to 9% in Ethiopia.

**Table 3:** Prevalence of Gestational Diabetes in Sub-Saharan Africa 1990-2011

Country	Author	Site	Sample size	Method	Prevalence GDM among women giving birth (%) (95% CI)
Ethiopia	Seyoum (1999) [42]	Rural	890	OGTT	3.7 (2.5 - 4.9)

Ethiopia	Hailu (1994) [44]	Rural/Urban	567	OGTT	9.2

South Africa	Mamabolo (2006) [43]	Rural	262	OGTT	1.5 (0.4 - 3.8)

Tanzania	Swai (1991) [45]	Rural	189	OGTT	0

South Africa	Ranchod (1991) [46]	Urban	1721	OGTT	3.8

#### Other Types of Diabetes

Several reviews describing diabetes trends in Africa report the occurrence of other forms of diabetes, namely *'Atypical African diabetes' *or *'Ketosis-prone atypical diabetes mellitus' *and *'Malnutrition-related diabetes' *or '*Tropical Diabetes' *[19,20,47]. However, beyond describing their existence and aetiology, no studies investigating the population prevalence of these forms of diabetes in the region were identified.

### Outcomes of diabetes

#### Diabetes mortality

Three studies investigated mortality in patients with diabetes in the region, two of which were conducted almost twenty years ago (Table 4). These studies revealed high mortality proportions, with 5-year mortality ranging from 4% - 57% [48-50]. 41% of individuals with Insulin-Dependent Diabetes Mellitus (IDDM) died within five years in a study in Tanzania, and half of these deaths were attributed to ketoacidosis [50]. Infection was another important cause of mortality, accounting for 48% of deaths in indeterminate diabetes cases, 32% in IDDM and 23% in Non-Insulin Dependent Diabetics (NIDDM) in the Tanzanian survey [50].

**Table 4:** Diabetes mortality studies in Sub-Saharan Africa from 1990

Country	Author (Year)	Diabetes Types	Sample (loss to follow up)	Mortality (%)		Mortality causes (deaths by cause/total deaths)
					
				5 year	20 year	
Ethiopia	Lester (1992) [49]	Type 1	431	4	37	-

South Africa	Gill (2005)[48]	Type 1	88 (39)	-	33	Renal failure (9/21) Hypoglycaemia (6/21) Ketoacidosis (2/21) Infection (2/21) Undetermined (2/21)

Tanzania	McLarty (1990) [50]	IDDM^1^	272	40.5	-	Leading causes: IDDM: 50% Ketoacidosis; NIDDM: 24% Cardiovascular & renal disease; Indeterminate: 48% infection.
		NIDDM^2^	825	19	-	
		Indeterminate type	153	57	-	

^1^IDDM: Insulin Dependent Diabetes Mellitus; ^2^NIDDM: Non Insulin Dependent Diabetes Mellitus.

#### The prevalence of chronic diabetes complications among persons with diabetes

Twenty-three studies on the prevalence of chronic complications of diabetes among persons with diabetes published from 1999-2011 were reviewed (see table 5) [51-74]. The recorded prevalence of retinopathy ranged from 7% in Kenya [63], to 63% in South Africa [67], neuropathy ranged from 27% in Cameroon [52] to 66% in Sudan [54], and the prevalence of microalbuminuria ranged from 10% in Tanzania [74] to 83% in Nigeria [73]. Some national variation was suggested, with recorded retinopathy rates ranging from 7% to 22% in Kenya [63,64].

**Table 5:** Cross-sectional studies on chronic complications of diabetes in Sub-Saharan Africa 1999-2011

Complication	Location	Author (year)	Sample	Setting	Type of diabetes	Prevalence (%)
**Neuropathy**	Cameroon	Ndip (2006) [52]	300	Hospital inpatient and outpatient clinics	N/A	27.3%
	Nigeria	Odusan (2008) [53]	108	Hospital outpatient clinic	Type 2	Cardiac Autonomic Neuropathy: 34.2%
	Sudan	Ahmed (2000) [54]	120	Hospital outpatient clinic	Type 1 & 2	Cardiac Autonomic Neuropathy: 40% Peripheral: 66%

**Foot ulcers**	Cameroon	Ndip (2006) [52]	300	Hospital inpatient and outpatient clinics	N/A	13%
		Kengne (2010) [55]	1841	Hospital inpatients	N/A	13%
	Nigeria	Ogbera (2006) [56]	1500	Hospital inpatients & outpatient clinic	Mixed	9.5%
	Tanzania	Gulam-Abbas (2002) [57]	627	Hospital inpatients	Mixed	15%

**Retinopathy**	Botswana	Mengesha (2006) [58]	401	Outpatient clinics	Mixed	9.2%
	Cameroon	Sobgnwi (1999) [59]	64	Hospital outpatient clinic	Mixed, non-proteinuric	37.5%
	Ethiopia	Seyoum (2001) [60]	340	Hospital outpatient clinic	Type 1 & 2	37.8%
	Ghana & Nigeria	Rotimi (2003) [61]	840	Hospital outpatient clinics	Type 2	17.9%
	Nigeria	Omolase 2010 [62]	100	Hospital outpatient clinic	Mixed	15%
	Kenya	Mwendwa (2005) [63]	100	Hospital outpatient clinic	Type 2	7%
	Kenya	Mwale (2007) [64]	96	Hospital outpatient clinic	Type 2	22%
	South Africa	Motala (2001) [65]	T1: 47 T2: 172	Hospital outpatient clinic	Type 1 & 2	Type 1: 53.2%, Type 2: 64.5%
	South Africa	Read & Cook (2007) [66]	248	Hospital outpatient clinic	Type 2	32.3%
	South Africa	Mash (2007) [67]	400	Outpatient clinics	Mixed	63%
	Tanzania	Majaliwa (2007)[68]	99	Hospital outpatient clinic	Type 1	22.68%
	Nigeria	Unuigbe (2001) 124	66	Hospital outpatient clinic	Type 1 & 2	23%

**Microalbuminuria**	Cameroon	Sobgnwi (1999) [59]	63	Hospital outpatient clinic	Mixed, non-proteinuric	53.1%
	Ghana	Eghan (2007) [71]	109	Hospital outpatient clinic	Mixed	43.1%
	Kenya	Wanjohi (2002) [51]	100	Hospital outpatient clinic	Type 2	Albuminuria: 26%
	Kenya	Mwendwa (2005) [63]	100	Hospital outpatient clinic	Type 2	25%
	Nigeria	Unuigbe (2001) [69]	66	Hospital outpatient clinic	Type 1 & 2	50%
	Nigeria	Agaba (2004) [72]	65	Hospital outpatient clinic	Type 2	49.2%
	Nigeria	Adetunji (2006) [73]	50	Hospital outpatient clinic	Type 2 > 5 yrs, non-proteinuric	83%
	Tanzania	Lutale (2007) [74]	T1: 91 T2: 153	Hospital outpatient clinic	Mixed	Type 1: 12%, Type 2: 9.8%
	Tanzania	Majaliwa (2007)[68]	99	Hospital outpatient clinic	Type 1	29.3%

**Coronary Heart Disease**	South Africa	Kalk (2007) [70]	744	Hospital outpatient clinic	Type 2	White: 23%, Black: 4%

#### Important infections and diabetes

Whilst epidemiological studies outside Sub-Saharan Africa have associated diabetes with infectious diseases of great importance in this region (see *Discussion *section), the literature review identified little epidemiological data on this association in Sub-Saharan Africa. A few smaller case-control studies have suggested that the prevalence of urinary tract infections is increased in diabetic patients in Sub-Saharan Africa, as observed in developed countries [75]. Several reviews have described the frequent occurrence of gangrene, infection and sepsis associated with diabetic foot ulcer disease [76] and with trauma to the hand (tropical diabetic hand syndrome) [77] in Sub-Saharan Africa.

#### Access to diabetes diagnosis and treatment

All of the type II prevalence surveys which recorded proportions of previously undiagnosed diabetes among participants who attended screening programmes found very high levels (≥40%). Proportions exceeded 50% in five surveys, and reached 100% in rural Guinea [24-27,29,33].

Beran et al surveyed the availability of diagnostic testing tools in a sample of healthcare settings in three countries, and found that in Mozambique urine glucose strips were available in just 18% of health facilities surveyed, ketone testing strips in 8% and blood glucose metres in 21%, whilst availability in Mali was 54%, 43% and 13% and in Zambia 61%, 54%, 49% [40,78].

Low levels of adequate glucose control in diagnosed diabetics were reported in several prevalence studies [24,68]. Only 27% of diagnosed type 2 diabetics receiving treatment in the Cameroon study had adequately controlled glucose levels [24]. Of 99 type 1 diabetics in the Tanzanian survey, only one person achieved good glucose control [68]. None of the 99 type 1 diabetics had the ability to monitor their glucose levels at home, and hospitals were unable to routinely do this [68]. A regular supply of insulin was unaffordable for many diabetics, with one month's insulin supply costing 19.6 days wages in Malawi [79] and 25% of the minimum wage in Tanzania [80]. One Sudanese study found that 65% of a families' annual household expenditure on health was spent on caring for a diabetic child [81]. Beran and Yudkin found that state interventions affected insulin price, reporting that an annual supply of insulin cost 5% of GDP in Mozambique, where it was subsidised by the government, whereas it cost 25% of GDP in Mali without subsidies [78]. One study investigated Insulin availability and reported that one of five hospitals and none of six health centres surveyed had a regular insulin supply[40].

### Economic Costs of Diabetes

Few studies were identified which investigated the cost of diabetes in the region. Kiriga et al (2009) estimated that the total economic cost (direct and indirect) of diabetes in the WHO's Africa region in 2000 was Int$25.51 billion (US$67.03 billion), or Int$3363 (US$8836) per person with diabetes per year [82] (numbers converted from International dollars to their equivalent value in US dollars [83]). Kiriga also estimated that the direct cost of treating diabetes in 2000 ranged from Int$876 (US$2302) to Int$1220.6 (US$3207) per person.

One study into the cost of caring for children with T1DM in Sudan found that the mean annual expenditure on diabetes care was US$283 per diabetic child, 36% of which was spent on insulin [81]. A Tanzanian study estimated that in 1989-1990 the total cost of outpatient care for all diabetic patients was US$2.7 million, of which insulin accounted for two-thirds of the expenditure, and total in-patient cost was US$1.25 million [84]. As diabetes care in Tanzania was provided free of charge to users this total cost of US$4 million was paid from the government health budget and accounted for 8% of the government's total health expenditure 1989-1990. A South African study investigated the cost of hyperglycaemic emergency admissions in South Africa over a two month period in 2005 and reported an average cost R5309, equivalent to US$712, per admission [85].

## Discussion

### Prevalence of diabetes

#### Prevalence of type 2 diabetes

The prevalence of T2DM appears to have increased considerably from that recorded in earlier (pre-1985) surveys conducted in the region, which found the prevalence in Sub-Saharan Africa was typically below 1%, with the exception of studies in South Africa (3.6%) [86] and the Ivory Coast (5.7% (Zmirou, D. 1979 thesis, reported in McLarty et al 1990 and Motala et al 2003)) [87,88]. However, many of these early studies may have underestimated prevalence, due to the use of low sensitivity screening methods and non-standardised diagnostic criteria [33,89,90].

Impaired Glucose Tolerance (IGT) and Impaired Fasting Glucose (IFG) are predictors of incident T2DM [91]. Thus, a high IGT prevalence alongside a low T2DM prevalence may indicate the early stage of a diabetes epidemic [92]. IGT prevalence suggests that the T2DM prevalence is likely to increase further in several countries in the region, including Cameroon, Ghana and Guinea (Table 1)...

Preventing obesity and increasing level of physical activity is important for reducing the onset of T2DM. Eight T2DM surveys measured the prevalence of obesity among participants, which ranged from 0.2% among rural males in Tanzania [34] to 21% among females in urban Cameroon [24]. The Ministry of Health study in Cameroon reported that controlling obesity and overweight levels would reduce diabetes by 15% in males and 13% in females [24]. The comparatively higher prevalence of T2DM recorded in urban areas was associated with a higher prevalence of obesity among the urban samples and a lower proportion reporting regular physical activity [23,34]. The projections that by 2025 70% of Africans will live in cities, with a regional annual urban growth rate of 4.5% [93], suggest that levels of obesity and T2DM diabetes will continue to rise in the region [19].

#### Prevalence of other types of diabetes

As there is very limited data available on other forms of diabetes in the region it is difficult to describe trends. Given the small number and limitations of existing studies, it is not clear whether the very low incidence rates of T1DM of 1-2 per 100,000 are reliable. All the existing studies based their estimates on previously diagnosed cases rather than population screening, and misdiagnosis and high community mortality may leave many cases unknown [20,39,68]. This is supported by the observation that T1DM was often first diagnosed when patients presented at healthcare facilities with acute diabetic complications [48,85]. Similarly, more studies measuring the prevalence of gestational diabetes are needed to determine whether the observed variation from 0 to 9% among pregnant women reflects true regional variation.

### Outcomes of diabetes

#### Mortality and chronic complications

The three studies investigating mortality following diabetes found high proportions, and these may have been underestimates or overestimates due to sampling only selected diagnosed diabetic patients accessing healthcare. More large-scale population-based studies are needed to explore whether the varying diabetes complication proportions in different countries reflect true variation. Small samples sizes and hospital-based recruitment limit the generalisability of these studies.

The observed high mortality in patients with diabetes and high prevalence of diabetes complications is likely to be a consequence of many late diagnosed and poorly controlled cases. Assessing the public health importance of diabetes demands an appreciation of the impact of diabetes on other diseases and population mortality, and in particular the benefits of well-controlled diabetes for averting costly cardiovascular and microvascular complications [7,94]. The total cost of these complications is likely to far outweigh the cost of effective primary and secondary prevention.

#### Infections

Potential associations between diabetes and important communicable diseases in the region, particularly tuberculosis and HIV, further complicate the pattern of increasing diabetes prevalence in Sub-Saharan Africa and the challenges posed on resource-constrained health systems. Newer studies primarily outside Sub-Saharan Africa provide evidence on such associations.

A recent meta-analysis of thirteen studies found that diabetes was associated with a 3.1 times elevated risk of tuberculosis [11], and a systematic review of 9 studies found that this increased risk varied from 1.5 to 7.8 [95]. Studies have suggested that 20% of smear-positive tuberculosis in India and 25% of tuberculosis in Mexico is attributable to diabetes [96,97]. The increased risk of tuberculosis associated with diabetes may be largely attributed to poor glycemic control [98,99]. Unfortunately, proper epidemiological studies of the diabetes-tuberculosis association from Africa are to our knowledge missing [100].

Meanwhile, the high prevalence of HIV and the roll-out of ART may increase the prevalence of diabetes risk factors and consequently diabetes incidence. Antiretroviral therapy (ART) for HIV, and to a small extent, HIV itself, is associated with an increased risk of developing the metabolic syndrome, which predisposes individuals to develop type 2 diabetes and cardiovascular disease [101]. A range of metabolic changes have been associated with ART, such as increased central obesity [101], increased insulin resistance [102], lipodystophy [103], and dislipidemia [104]. These changes have also been associated, although to a much lesser extent, with untreated HIV[102]. One review found that prevalence of metabolic syndrome among patients on ART ranged between 18 and 33% [105].

Evidence from other countries of the associations between diabetes and a range of other severe infectious diseases also deserves consideration. Type 2 diabetes is associated with a 25-75% increased risk of pneumonia and pneumococcal bacteremia leading to hospitalisation [106-111], and longer duration of diabetes, diabetes complications, and poor long-term glycemic control increase the risk [108,112]. Diabetes increases the risk of developing severe sepsis, with one study reporting a 2.5-fold increased risk for hospitalization with sepsis in diabetic individuals compared to the general population [110]. Patients with diabetes mellitus have a two to three fold increased risk of bacteremia and sepsis originating from the urinary tract compared with those without diabetes [113]. Among 11 patients newly admitted with type 1 diabetes at a teaching hospital in Nigeria, 9 (82%) presented with urinary tract infection, malaria, or recurrent boils [114]. Bacteremia risk due to hemolytic streptococci and staphylococci is also increased two to three-fold in patients with diabetes [115-117], and often originates from wound infections, which are an important health problem in the region [20].

Projections of future diabetes trends have not considered the potential impact of these associations which could affect the future diabetes burden. Consideration of the associations between diabetes and the other major communicable diseases in the region has been notably absent from the literature - both peer reviewed and grey literature. It is vital that awareness of these associations is promoted so that complementary and integrated programmes in these disease areas can be planned [118].

#### Access to diagnosis and treatment

The high rates of undiagnosed and uncontrolled diabetes recorded highlight the presence of significant barriers to accessing diagnosis and treatment. The high rates of undiagnosed diabetes suggest that existing screening practices in the region are not effective. Given the reports that health centres lack the necessary diagnostic tools it is also likely that screening for diabetes is not routinely performed.

Several important challenges to accessing diagnosis and treatment have been identified in the literature: the high financial cost of treatment, particularly insulin; the limited availability of diagnostic tools, treatment and glucose monitoring equipment; and a low awareness of diabetes among healthcare professionals which was reported by some authors[40]. Other important barriers may exist that have not yet been identified, as few studies have focused on this issue, and more information on the comparative importance of these factors is necessary to effectively target any interventions.

### Economic Impacts of Diabetes

Diabetes is an expensive disease, especially when the cost of complications, including the many diseases where diabetes is an underlying causal factor, is considered. Kiriga's study highlighted the vast expense of treating diabetes in the WHO's African region; however the cost of complications were excluded from the study, and therefore this is a significant underestimate of total cost of diabetes. Kiriga estimated that the direct cost of treating diabetes in 2000 ranged from Int$876 (US$ 2302) to Int$1220.6 (US$3207) per person. Even at this level of direct cost, there is a significant discrepancy between the cost and available expenditure, as the International Diabetes Federation (IDF) has estimated that in 2010 national funding for the healthcare of diabetics in Africa is just US$111 per person, which already amounts to 7% of national healthcare expenditure [119].

With limited national funding, individual patients and their families may have to spend significant proportions of their income on treatment for diabetes, a level of expenditure that may not be sustainable or affordable. The Sudanese study found that families spend an average of US$283 per year caring for their diabetic child, which amounted to 65% of the family's annual expenditure on health. It is possible that in this scenario other health needs are overlooked in order to devote over 50% of annual health expenditure to the one member of the family with diabetes.

The burden of T2DM is disproportionately borne by people of working age [120], which is also the age-group most profoundly affected by HIV in this region. Reducing the economic activity of this group through disease and disability affects both household and national economies. Diabetes therefore not only imposes considerable costs of treatment on families, it also hinders their ability to pay for this treatment through the loss of income of the diabetic member. At a national level an increasing prevalence of diabetes among the economically active, and the high prevalence of diabetic complications and low survival rates, will negatively impact economic development, and in turn the health budget.

Information on the cost of diabetes, including the cost of the complications, is critical for policymakers to highlight the importance of introducing early and cost effective interventions for both primary and secondary prevention.

## Conclusion

With increasing prevalence and interactions with other diseases, including the major communicable diseases of the region, diabetes is becoming a pressing public health problem for Sub-Saharan Africa. If effective interventions are implemented in the near-future it may be possible to avert much of this burden, as primary prevention and treatment can reduce the incidence of both diabetes and a range of related diseases where diabetes is a causal factor. However, establishing timely and effective integrated diabetes programmes in the region requires a shift in current public health priorities, and this requires a much better evidence base - both to highlight the scale of the problem and the areas for intervention. Below are a set of recommendations for necessary action in order to address some of the knowledge gaps identified in this article. As identified in the WHO 2008-2013 Action Plan for the Global Strategy on Noncommunicable Diseases, these tasks require comprehensive efforts from multiple stakeholders, including countries, international organisations, academic institutions, civil society and the private sector.

### Recommendations

1. Countries, with the assistance from academic institutions, should ensure that local diabetes prevalence and incidence data are collected, for example through the recurring Demographic and Health Surveys, to increase the availability of good information on current epidemiological trends.

2. Academic institutions should collect regional and country-specific data on mortality, morbidity, costs, and access to diagnosis and care.

3. Under the leadership of intergovernmental agencies such as the World Health Organization and the World Bank, models should be developed to assess the public health impact of diabetes in relation to other important diseases, to enable informed prioritisation of available health funding at country level.

4. International agencies, civil society and the private sector should join forces and use their expertise and network to promote awareness of the interactions between diabetes and key communicable diseases in Sub-Saharan Africa, to inform the development of integrated and complimentary service delivery programmes and health policies.

5. International agencies and civil society should establish peer-learning and experience-sharing discussion forums to promote development of feasible and cost-effective strategies and solutions for management and control of diabetes in Sub-Saharan Africa.

## Competing interests

VH undertook this review as a consultant for Novo Nordisk A/S. NL and OH are both employees of Novo Nordisk A/S.

## Authors' contributions

NL and OH conceived the study and wrote the analysis plan. VH conducted the literature review and analysis and wrote the first draft manuscript. NL, RWT and OH reviewed the draft manuscript, provided critical comments and suggested additional analyses. VH finalised the manuscript which was subsequently approved by all authors.

## Pre-publication history

The pre-publication history for this paper can be accessed here:

http://www.biomedcentral.com/1471-2458/11/564/prepub

## Supplementary Material

Additional file 1 **Annex 1: Flow Diagram of Studies Reviewed**. This is a flow diagram of the studies reviewed in this systematic review. Click here for file

Additional file 2 **Annex 2: Keyword search terms**. This describes the keywords used to perform the literature search. Click here for file
