# Supplementary material for: Diabetes in Sub Saharan Africa 1999-2011: Epidemiology and public health implications. a systematic review
Source: BMC Public Health. 2011 Jul 14;11:564. doi: 10.1186/1471-2458-11-564 (PMC3156766; doi:10.1186/1471-2458-11-564)
Supplement: Additional file 1 — Annex 1: Flow Diagram of Studies Reviewed. This is a flow diagram of the studies reviewed in this systematic review. [file 1471-2458-11-564-S1.DOC]

**Annex 1: Flow Diagram of Studies reviewed**

| **PubMed keyword search** | **Hits** |
| --- | --- |
| 1. “Diabetes” OR “Diabetes Mellitus” | 361486 |
| 1. “Africa south of the Sahara OR...” (see Annex 2) | 191688 |
| 1. #1 AND #2 | 2545 |
| 1. Limited to 01/01/1999 to 31/03/2011, limited to written in English and Humans | 1102 |

Outside region, Other disease, Genetic research, Microbiological research, Description of diabetes pathogenesis.

**627 Excluded**

**Type 2 Prevalence Studies**

**16**

**Type 1 Prevalence studies**

**4**

**Gestational Diabetes prevalence studies**

**5**

**Prevalence of complications**

**23**

N<50, hospital setting, duplicates, no measure of diabetes prevalence, DM prevalence not a primary aim, measuring prevalence of diabetes risk factors, measuring diabetes as a risk factor for other diseases, case reports.

14

2 from hand-searching (MOH surveys)

112 papers reviewed for prevalence/incidence diabetes

**87 Excluded**

164 papers reviewed for prevalence of complications

199 papers reviewed for information on costs, burden, and interactions with infectious diseases (e.g. HIV/AIDS and TB)

2

2

2 from hand-searching

3 from hand-searching

1 Review

N<50, setting too selective, sample too selective, studies on prevalence of CVD risk factors among diabetics, management and screening options, or studies on prevalence of deafness, periodontal problems, mental health problems among diabetics

**140 Excluded**

**Mortality Studies**

**3**

2 from hand-searching

1

6 Reviews
