# Supplementary material for: Diabetes in Sub Saharan Africa 1999-2011: Epidemiology and public health implications. a systematic review
Source: BMC Public Health. 2011 Jul 14;11:564. doi: 10.1186/1471-2458-11-564 (PMC3156766; doi:10.1186/1471-2458-11-564)
Supplement: Additional file 2 — Annex 2: Keyword search terms. This describes the keywords used to perform the literature search. [file 1471-2458-11-564-S2.DOC]

**Annex 2: Keyword search terms**

| **Search** | **Search Terms** | **Hits** |
| --- | --- | --- |
| 1 | Diabetes OR Diabetes Mellitus | 361486 |
| 2 | "africa south of the sahara"[MeSH Terms] OR ("africa"[All Fields] AND "south"[All Fields] AND "sahara"[All Fields]) OR "africa south of the sahara"[All Fields] OR ("sub"[All Fields] AND "saharan"[All Fields] AND "africa"[All Fields]) OR "sub saharan africa"[All Fields] OR Angola OR Benin OR Botswana OR Burkina Faso OR Burundi OR Cameroon OR Central African Republic OR Chad OR Congo OR Comoros OR Cote d'Ivoire OR Democratic Republic of the Congo OR Equatorial Guinea OR Eritrea OR Ethiopia OR Gabon OR Gambia OR Ghana OR Guinea-Bissau OR Kenya OR Lesotho OR Liberia OR Madagascar OR Malawi OR Mali OR Mauritania OR Mozambique OR Namibia OR Niger OR Nigeria OR Republic of Congo OR Rwanda OR Sao Tome and Principe OR Senegal OR Sierra Leone OR Somalia OR South Africa OR Sudan OR Swaziland OR Tanzania OR Togo OR Uganda OR United Republic of Tanzania OR Zambia OR Zimbabwe | 191688 |
| 3 | 1 + 2 | 2545 |
| 4 | Limit to dates 01/01/1990 to 31/03/2011, in English and in Humans | 1102 |
